# Supplementary material for: Rhythmic astrocytic GABA production synchronizes neuronal circadian timekeeping in the suprachiasmatic nucleus
Source: EMBO J. 2024 Dec 2;44(2):356–81. doi: 10.1038/s44318-024-00324-w (PMC11731042; doi:10.1038/s44318-024-00324-w)
Supplement: Supplementary file 1 — Appendix [file 44318_2024_324_MOESM1_ESM.pdf]

Appendix for

Rhythmic astrocytic GABA production synchronizes neuronal circadian timekeeping in the suprachiasmatic nucleus

Natalie Ness[1,2], Sandra Díaz-Clavero[1,2], Marieke MB Hoekstra[1,2] and Marco Brancaccio[1,2*]

Affiliations:

[1]Department of Brain Science, Imperial College London, United Kingdom

[2]UK Dementia Research Institute at Imperial College London, London, United Kingdom.

*Corresponding author. Email: m.brancaccio@imperial.ac.uk

Table of contents

1. Appendix Figure S1 Page 2
2. Appendix Figure S2 Page 3
3. Appendix Table S1 Page 4

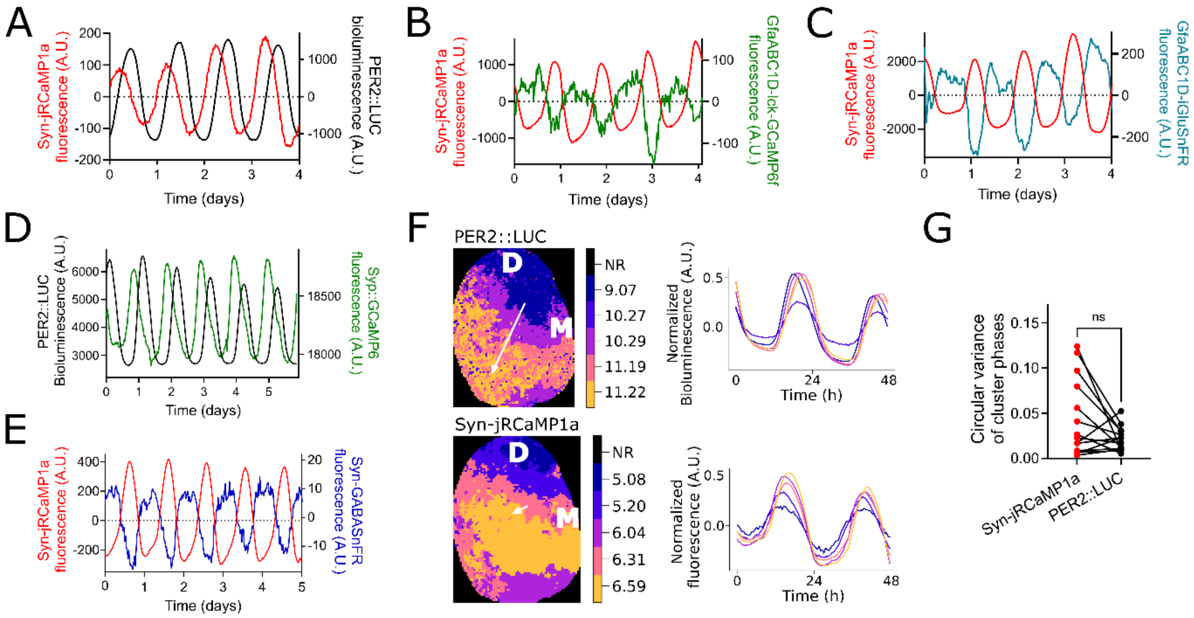

Appendix Figure S1: **Circadian oscillations of neuronal and astrocytic reporters in organotypic SCN slices and spatiotemporal phase waves of clock gene expression and neuronal calcium.** Representative traces showing circadian oscillations of genetically encoded reporters used in this manuscript: (A) Syn-jRCaMP1a and PER2::LUC, (B) Syn-jRCaMP1a and GfaABC1D-lck-GCaMP6f, (C) Syn-jRCaMP1a and GfaABC1D-iGluSnFR, (D) PER2::LUC and Syp::GCaMP6s, (E) Syn-jRCaMP1a and Syn-GABASnFR. All reporters plotted within the same graph were monitored simultaneously within the same SCN. (F) Representative circadian phase map of PER2::LUC (top) and Syn-jRCaMP1a (bottom) in SCN slices, showing phase progression wave from the dorsomedial to ventrolateral SCN. One SCN is shown (top=dorsal-D, right=medial-M), showing dorsomedial to ventrolateral spatiotemporal progression of PER2:LUC expression and neuronal calcium levels. The white vector indicates the direction phase progression (see Methods). The color bar shows the circadian phase of each cluster, with corresponding time series of the clusters shown to the right. NR = non-rhythmic. (G) Inter-cluster phase dispersal, as measured by circular variance of phases, of co-detected PER2::LUC and Syn-jRCaMP1a. Each dot represents one SCN slice, N=14. Two-tailed paired t-test. ns=non-significant.

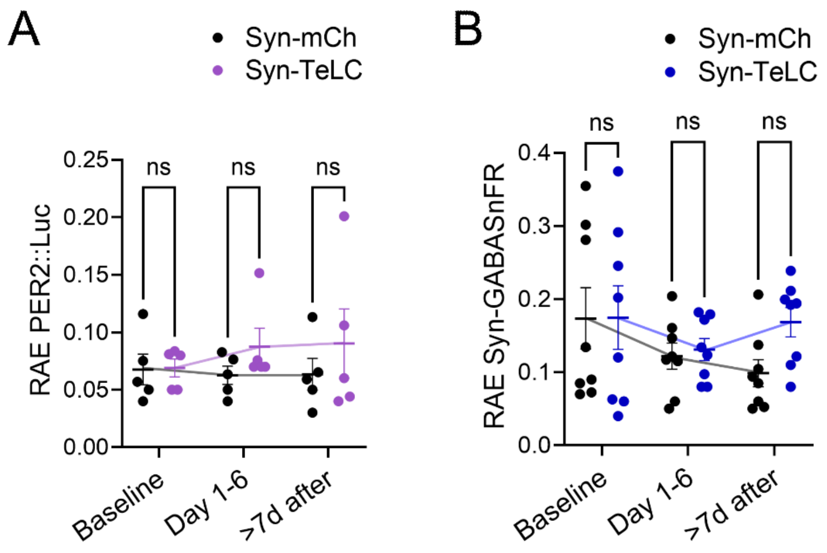

Appendix Figure S2: Disrupting synaptic activity via genetic expression of tetanus toxin light chain does not significantly alter PER2::LUC or Syn-GABASnFR rhythm robustness. Relative amplitude error (RAE) of rhythms of PER2::LUC (A) (N=5 per condition) and Syn-GABASnFR (B) (N=8 per condition) before and after transduction with Syn-TeLC or Syn-mCherry control. All data mean ± SEM. Statistical test: two-way ANOVA with post-hoc Šidák's test, ns=non-significant.

Appendix Table S1: Detailed statistical test results.

Figure	Statistical Test	Mean difference (p-value)	Multiple comparisons	p-value
1D (left)	Paired two-tailed t-test	0.0105		
1D (right)	Kolmogorov-Smirnov test	0.0079		
1F (left)	Paired two-tailed t-test	0.0477		
1F (right)	Kolmogorov-Smirnov test	0.0025		
2A (left)	Mixed-effects model with Tukey's multiple comparison's test	0.6388	PER2::Luc vs. Syn-jRCaMP1a	0.9984
			PER2::Luc vs. Syn-GABASnFR	0.986
			PER2::Luc vs. Syp::GCaMP6s	0.5739
			Syn-jRCaMP1a vs. Syn-GABASnFR	0.9979
			Syn-jRCaMP1a vs. Syp::GCaMP6s	0.6858
			Syn-GABASnFR vs. Syp::GCaMP6s	0.7315
2A (right)	Mixed-effects model with Tukey's multiple comparison's test	0.1679	PER2::Luc vs. Syn-jRCaMP1a	0.981
			PER2::Luc vs. Syn-GABASnFR	0.3751
			PER2::Luc vs. Syp::GCaMP6s	0.9668
			Syn-jRCaMP1a vs. Syn-GABASnFR	0.1863
			Syn-jRCaMP1a vs. Syp::GCaMP6s	0.9963
			Syn-GABASnFR vs. Syp::GCaMP6s	0.5154
2E (top)	Paired two-tailed t-test	0.4801		
2E (bottom)	Paired two-tailed t-test	0.0079		
2F (top)	Kolmogorov-Smirnov test	0.357		
2F (bottom)	Kolmogorov-Smirnov test	0.00236		
2G	Mixed-effects model with Tukey's multiple comparison's test	<0.0001	Syn-jRCaMP1a vs. PER2::LUC	0.0539
			Syn-jRCaMP1a vs. GfaABC1D-lck-GCaMP6f	0.001
			Syn-jRCaMP1a vs. GfaABC1D-GluSnFr	0.0157
			Syn-jRCaMP1a vs. Syp::GCaMP6s	0.841
			Syn-jRCaMP1a vs. Syn-GABASnFR	<0.0001
			PER2::LUC vs. GfaABC1D-lck-GCaMP6f	0.2147
			PER2::LUC vs. GfaABC1D-GluSnFr	0.5099
			PER2::LUC vs. Syp::GCaMP6s	0.0937
			PER2::LUC vs. Syn-GABASnFR	0.1862
			GfaABC1D-lck-GCaMP6f vs. GfaABC1D-GluSnFr	>0.9999
			GfaABC1D-lck-GCaMP6f vs. Syp::GCaMP6s	0.0035
			GfaABC1D-lck-GCaMP6f vs. Syn-GABASnFR	0.9936
			GfaABC1D-GluSnFr vs. Syp::GCaMP6s	0.0143
			GfaABC1D-GluSnFr vs. Syn-GABASnFR	0.9997
			Syp::GCaMP6s vs. Syn-GABASnFR	0.0029
2H	Rayleigh's test of uniformity		PER2::LUC	<0.0001
			Syn-jRCAMP1a	<0.0001

Figure	Statistical Test	Mean difference (p-value)	Multiple comparisons	p-value
			Syp::GCaMP6s	0.0151
			GfaABC1D-lck-GCaMP6f	0.753
			GfaABC1D-iGluSnFR	0.763
			Syn-GABASnFR	0.0870
3C	Unpaired two-tailed t-test	0.0008		
3E (left)	Two-way ANOVA with Tukey's multiple comparisons test	Time 0.003 Treatment 0.0598 Interaction 0.023	Day1-6: Syn-mCh vs Syn-TeLC	0.1746
			>7d after: Syn-mCh vs Syn-TeLC	0.0526
			Syn-mCh: BSL vs Day1-6	0.5774
			Syn-mCh: BSL vs >7d after	0.8346
			Syn-mCh: Day1-6 vs >7d after	0.7994
			Syn-TeLC: BSL vs Day1-6	0.0152
			Syn-TeLC: BSL vs >7d after	0.0002
			Syn-TeLC: Day1-6 vs >7d after	0.0682
3E (right)	Two-way ANOVA with Tukey's multiple comparisons test	Time 0.0172 Treatment 0.5818 Interaction 0.2866	Day1-6: Syn-mCh vs Syn-TeLC	0.8603
			>7d after: Syn-mCh vs Syn-TeLC	0.3319
			Syn-mCh: BSL vs Day1-6	0.7661
			Syn-mCh: BSL vs >7d after	0.042
			Syn-mCh: Day1-6 vs >7d after	0.0249
			Syn-TeLC: BSL vs Day1-6	0.6851
			Syn-TeLC: BSL vs >7d after	0.6185
			Syn-TeLC: Day1-6 vs >7d after	0.6302
3F (left)	Two-way ANOVA with Šídák's multiple comparisons test	Time 0.7763 Treatment 0.5737 Interaction 0.8666	Baseline	0.9532
			Day 1-6	0.9992
			>7d after	0.8297
3F (right)	Two-way ANOVA with Šídák's multiple comparisons test	Time 0.1174 Treatment 0.8601 Interaction 0.3657	Baseline	0.9922
			Day 1-6	0.8653
			>7d after	0.8062
3G	Two-way ANOVA with Šídák's multiple comparisons test	Time <0.0001 Treatment 0.76064 Interaction 0.7245	Baseline	0.9896
			Day 1-6	0.9904
			>7d after	0.9178
3L (top)	Unpaired two-tailed t-test	0.0338		
3L (bottom)	Unpaired two-tailed t-test	0.2589		
4F (left)	Two-way ANOVA with Šídák's multiple comparisons test	Gene <0.0001 Cell type <0.0001 Interaction <0.0001	Gad2 HY Astros vs. SCN Neurons	<0.0001
			Gad2 HY Astros vs. HY GABA Neurons	<0.0001
			Gad2 SCN Neurons vs. HY GABA Neurons	<0.0001
			Gad1HY Astros vs. SCN Neurons	<0.0001
			Gad1 HY Astros vs. HY GABA Neurons	<0.0001

Figure	Statistical Test	Mean difference (p-value)	Multiple comparisons	p-value
			Gad1SCN Neurons vs. HY GABA Neurons	0.5416
4F (right)	Two-way ANOVA with Šídák's multiple comparisons test	Gene <0.0001 Cell type <0.0001 Interaction <0.0001	Maob HY Astros vs. SCN Neurons	<0.0001
			Maob HY Astros vs. HY GABA Neurons	<0.0001
			Maob SCN Neurons vs. HY GABA Neurons	<0.0001
			Aldh1a1 HY Astros vs. SCN Neurons	<0.0001
			Aldh1a1 HY Astros vs. HY GABA Neurons	<0.0001
			Aldh1a1 SCN Neurons vs. HY GABA Neurons	0.9503
			Aldh1a1 HY Astros vs. SCN Neurons	0.6098
			Aldh1a1 HY Astros vs. HY GABA Neurons	<0.0001
			Aldh1a1 SCN Neurons vs. HY GABA Neurons	<0.0001
			Aldh1a1 HY Astros vs. SCN Neurons	0.0046
			Aldh1a1 HY Astros vs. HY GABA Neurons	<0.0001
			Aldh1a1 SCN Neurons vs. HY GABA Neurons	0.9587
4H (left)	Two-way ANOVA with Šídák's multiple comparisons test	Gene <0.0001 Cell type <0.0001 Interaction 0.6945	Gad1	<0.0001
			Gad2	<0.0001
4H (right)	Two-way ANOVA with Šídák's multiple comparisons test	Gene <0.0001 Cell type <0.0001 Interaction 0.6945	Maob	<0.0001
			Aldh1a1	<0.0001
			Sirt2	<0.0001
4I	eJTK Cycle rhythmcity test with BH correction		Gad1 Neurons	0.148
			Gad2 Neurons	0.0614
			Gad1 Astrocytes	0.2281
			Gad2 Astrocytes	0.2283
4J	eJTK Cycle rhythmcity test with BH correction		Aldh1a1 Astrocytes	0.0218
			Maob Astrocytes	0.0653
			Aldh1a1 Neurons	0.1278
			Maob Neurons	0.1780
5C	Two-way ANOVA with Šídák's multiple comparisons test	Time 0.0007 Treatment 0.0331 Interaction 0.3944	Baseline	0.1466
			Treatment	0.0303
5E	Two-way ANOVA with Šídák's multiple comparisons test	Time 0.8697 Treatment 0.233 Interaction 0.4605	Baseline	0.2928
			Treatment	0.8636
5F	Unpaired two-tailed t-test	0.1378		
5G		Time 0.2401	BSL DMSO vs. Sel	0.6991

Figure	Statistical Test	Mean difference (p-value)	Multiple comparisons	p-value
	Two-way ANOVA with Šídák's multiple comparisons test	Treatment 0.299 Interaction 0.7141	Treatmen DMSO vs. Sel	0.4667
			DMSO Baseline vs. Treatment	0.7898
			Selegiline Baseline vs. Treatment	0.4982
5H	Two-way ANOVA with Šídák's multiple comparisons test	Time 0.8199 Treatment 0.1272 Interaction 0.0333	BSL DMSO vs. Sel	0.8506
			Treatmen DMSO vs. Sel	0.0176
			DMSO Baseline vs. Treatment	0.2603
			Selegiline Baseline vs. Treatment	0.1846
5J	Two-way ANOVA with Šídák's multiple comparisons test	Time 0.3076 Treatment 0.2704 Interaction 0.774	Baseline	0.559
			Treatment	0.4258
5K	Unpaired two-tailed t-test	0.0341		
5L	Two-way ANOVA with Šídák's multiple comparisons test	Time 0.6845 Treatment 0.1217 Interaction 0.0474	BSL DMSO vs. Sel	0.764
			Treatmen DMSO vs. Sel	0.0443
			DMSO Baseline vs. Treatment	0.3304
			Selegiline Baseline vs. Treatment	0.19
5M	Two-way ANOVA with Šídák's multiple comparisons test	Time 0.1341 Treatment 0.0985 Interaction 0.0599	BSL DMSO vs. Sel	0.9069
			Treatmen DMSO vs. Sel	0.0214
			DMSO Baseline vs. Treatment	0.9059
			Selegiline Baseline vs. Treatment	0.0657
6C	Unpaired two-tailed t-test	0.0085		
6D	Two-way mixed effects model with Šídák's multiple comparisons test	Time 0.028 Treatment 0.0072 Interaction 0.0034	Treatment	0.0092
			Washout	0.1104
6E	Two-way mixed effects model with Šídák's multiple comparisons test	Time 0.2015 Treatment 0.3925 Interaction 0.0955	Baseline	0.9376
			Treatment	0.0586
			Washout	0.9994
6F	Two-way mixed effects model with Šídák's multiple comparisons test	Time 0.4417 Treatment 0.0821 Interaction 0.8391	Baseline	0.2452
			Treatment	0.308
			Washout	0.2849
6H	Unpaired two-tailed t-test	0.4235		
6I	Two-way mixed effects model with Šídák's multiple comparisons test	Time 0.4973 Treatment 0.2306 Interaction 0.3265	Treatment	0.1574
			Washout	0.9935
6J		Time 0.1287	Baseline	0.3531

Figure	Statistical Test	Mean difference (p-value)	Multiple comparisons	p-value
	Two-way mixed effects model with Šídák's multiple comparisons test	Treatment 0.0556 Interaction 0.3454	Treatment	>0.9999
			Washout	0.0364
6L	Unpaired two-tailed t-test	0.7328		
6M	Two-way mixed effects model with Šídák's multiple comparisons test	Time 0.3212 Treatment 0.1194 Interaction 0.3143	Treatment	0.4575
			Washout	0.0591
6N	Two-way mixed effects model with Šídák's multiple comparisons test	Time 0.3719 Treatment 0.058 Interaction 0.6464	Baseline	0.2156
			Treatment	0.6916
			Washout	0.6482
7B	Two-way ANOVA with Šídák's multiple comparisons test	Time 0.0106 Treatment 0.3371 Interaction 0.132	BSL DMSO vs A37	0.8237
			Day 1-3 DMSO vs A37	0.3231
			DMSO BSL vs Day 1-3	0.5389
			A37 BSL vs Day 1-3	0.0085
7D	Two-way ANOVA with Šídák's multiple comparisons test	Time 0.3132 Treatment 0.6054 Interaction 0.3885	BSL DMSO vs A37	0.6049
			Day 1-3 DMSO vs A37	0.9998
7E	Nested two-tailed t-test	0.0807		
7F	Kolmogorov-Smirnov test		DMSO BSL vs treatment	<0.0001
			A37 BSL vs treatment	<0.0001
7G	Kolmogorov-Smirnov test		DMSO vs A37 treatment	<0.0001
7I	Two-way ANOVA with Šídák's multiple comparisons test	Time 0.0003 Treatment 0.0018 Interaction 0.0011	BSL DMSO vs A37	0.8555
			Day 1-3 DMSO vs A37	<0.0001
			DMSO BSL vs Day 1-3	0.7898
			A37 BSL vs Day 1-3	<0.0001
7K	Two-way ANOVA with Šídák's multiple comparisons test	Time 0.1389 Treatment 0.6074 Interaction 0.6043	BSL DMSO vs A37	0.9124
			Day 1-3 DMSO vs A37	0.7743
7L	Nested two-tailed t-test	0.5504		
7F	Kolmogorov-Smirnov test		DMSO BSL vs treatment	0.102
			A37 BSL vs treatment	<0.0001
7G	Kolmogorov-Smirnov test		DMSO vs A37 treatment	<0.0001
EV1C	Two-way ANOVA with Šídák's multipl	Region 0.3376	NucBlue D vs. V	0.8751
			D vs. M	>0.9999

Figure	Statistical Test	Mean difference (p-value)	Multiple comparisons	p-value
	e comparisons test	Reporter 0.0194 Interaction 0.9409	D vs. L	0.9787
			V vs. M	0.989
			V vs. L	>0.9999
			M vs. L	0.9965
			Syn-jRCaMP1a D vs. V	0.9924
			D vs. M	0.9674
			D vs. L	0.9798
			V vs. M	0.9997
			V vs. L	0.9989
			M vs. L	>0.9999
			GfaABC1D-lck-GCaMP6f D vs. V	0.9926
			D vs. M	0.9444
			D vs. L	0.9581
			V vs. M	>0.9999
			V vs. L	0.9992
			M vs. L	0.9306
			GFAP D vs. V	0.9663
			D vs. M	0.999
			D vs. L	0.9976
			V vs. M	0.8979
			V vs. L	0.935
			M vs. L	>0.9999
EV1E (top)	Two-way ANOVA with Šídák's multiple comparisons test	Region 0.9797 Reporter 0.0387 Interaction 0.9502	Syn-jRCaMP1a Anterior vs. Medial	>0.9999
			Anterior vs. Posterior	0.9822
			Medial vs. Posterior	0.9831
			gfaABC1D-lck-GCaMP6f Anterior vs. Medial	0.9997
			Anterior vs. Posterior	0.9993
			Medial vs. Posterior	>0.9999
EV1E (bottom)	Watson-Williams test	0.341077		
EV1F (top)	Two-way ANOVA with Šídák's multiple comparisons test	Region 0.3723 Reporter 0.0458 Interaction 0.2417	Syn-jRCaMP1a	0.2541
			gfaABC1D-iGluSnFR	0.9724
EV1F (bottom)	Watson-Williams test	0.067746		
EV1G (top)	Two-way ANOVA with Šídák's multiple comparisons test	Region 0.8078 Reporter 0.0111 Interaction 0.8055	Syn-jRCaMP1a Anterior vs. Medial	>0.9999 0.8471
			Anterior vs. Posterior	0.783
			Medial vs. Posterior	>0.9999
			gfaABC1D-lck-GCaMP6f Anterior vs. Medial	0.9983
			Anterior vs. Posterior	0.9984
			Medial vs. Posterior	>0.9999
EV1G (bottom)	Watson-Williams test	0.628638		
EV2D	Paired two-tailed t-test	0.0013		

Figure	Statistical Test	Mean difference (p-value)	Multiple comparisons	p-value
EV2E	Kolmogorov-Smirnov test	0.00397		
EV2G	Paired two-tailed t-test	0.0076		
EV3H	Two-way ANOVA with Šídák's multiple comparisons test	Gene <0.0001 Cell type <0.0001 Interaction <0.0001	Slc6a11 (GAT3)	<0.0001
			Slc6a1 (GAT1)	<0.0001
			Best1	<0.0001
EV3I	Two-way ANOVA with Šídák's multiple comparisons test	Gene <0.0001 Cell type <0.0001 Interaction <0.0001	Slc6a11 (GAT3)	<0.0001
			Slc6a1 (GAT1)	<0.0001
			Best1	0.0038
EV3J	eJTK Cycle rhythmicity test with BH correction		Slc6a11 Neurons	0.28569
			Slc6a11 Astrocytes	0.00444
EV4B	Two-way ANOVA with Šídák's multiple comparisons test	Reporter 0.0008 Treatment 0.0687 Interaction 0.0193	GFAP	0.2889
			Gfap-mCherry-Cre	0.9979
			GABA	0.0053
EV4C	Unpaired two-tailed t-test	0.3505		
EV4E	Two-way ANOVA with Šídák's multiple comparisons test	Time 0.7159 Treatment 0.38 Interaction 0.7248	BSL DMSO vs Selegiline	0.5616
			Treatment DMSO vs Selegiline	0.7954
			DMSO BSL vs Treatment	0.8347
			Selegiline BSL vs Treatment	>0.9999
EV4F	Unpaired two-tailed t-test	0.0947		
EV4G	Two-way ANOVA with Šídák's multiple comparisons test	Time 0.1699 Treatment 0.8814 Interaction 0.6577	BSL DMSO vs Selegiline	0.8955
			Treatment DMSO vs Selegiline	0.9765
			DMSO BSL vs Treatment	0.3255
			Selegiline BSL vs Treatment	0.7471
EV4H	Two-way ANOVA with Šídák's multiple comparisons test	Time 0.0352 Treatment 0.4783 Interaction 0.3145	BSL DMSO vs Selegiline	0.4616
			Treatment DMSO vs Selegiline	0.9808
			DMSO BSL vs Treatment	0.498
			Selegiline BSL vs Treatment	0.0834
EV5B	One-way ANOVA with Šídák's multiple comparisons test	0.0180	DMSO vs. 10µM	0.4455
			DMSO vs. 25µM	0.0161
			DMSO vs. 50µM	0.0233
EV5C	Two-way mixed effects model with Šídák's multiple comparisons test	Time 0.009 Treatment 0.0065 Interaction 0.1432	Treatment DMSO vs 10uM	0.8599
			DMSO vs 25uM	0.0206
			DMSO vs 50uM	0.0052
			10uM vs 25uM	0.4209
			10uM vs 50uM	0.1023

Figure	Statistical Test	Mean difference (p-value)	Multiple comparisons	p-value
			25uM vs 50uM	0.8308
			Washout<br>DMSO vs 10uM	0.958
			DMSO vs 25uM	0.9943
			DMSO vs 50uM	0.055
			10uM vs 25uM	0.5053
			10uM vs 50uM	0.004
			25uM vs 50uM	0.0477
			Syn-GABASnFR DMSO Treatment vs. Washout	0.9791
			Syn-GABASnFR 10uM Treatment vs. Washout	0.0214
			Syn-GABASnFR 25uM Treatment vs. Washout	0.0022
			Syn-GABASnFR 50uM Treatment vs. Washout	0.4788
S1G	Paired two-tailed t-test	0.0583		
S2A	Two-way ANOVA with Šídák's multiple comparisons test	Time 0.6352<br>Treatment 0.3814<br>Interaction 0.5059	Baseline	0.9997
			Day 1-6	0.5169
			>7d after	0.8356
S2B	Two-way ANOVA with Šídák's multiple comparisons test	Time 0.0708<br>Treatment 0.4385<br>Interaction 0.187	Baseline	>0.9999
			Day 1-6	0.974
			>7d after	0.0668
