# Supplementary material for: Rhythmic astrocytic GABA production synchronizes neuronal circadian timekeeping in the suprachiasmatic nucleus
Source: EMBO J. 2024 Dec 2;44(2):356–81. doi: 10.1038/s44318-024-00324-w (PMC11731042; doi:10.1038/s44318-024-00324-w)
Supplement: Supplementary file 3 — Movie EV1 [file 44318_2024_324_MOESM3_ESM.zip › MovieEV1_zip/MovieEV1_legend.docx]

**
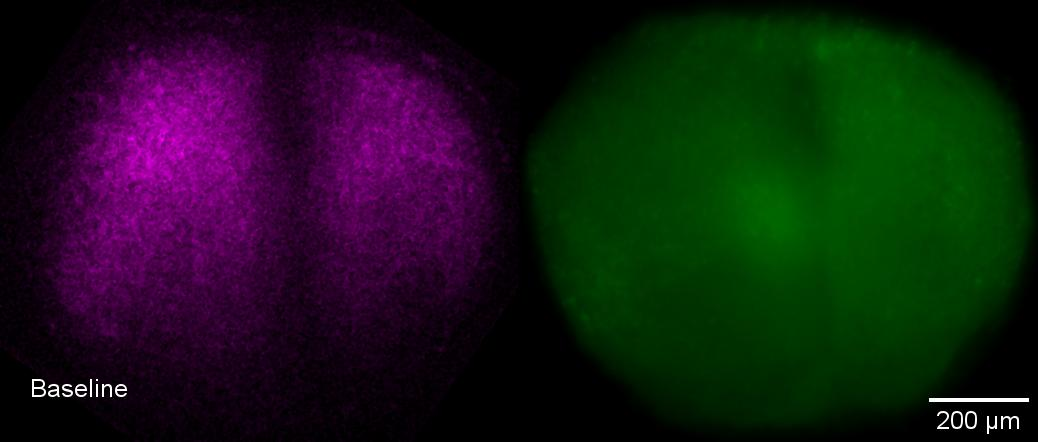
**

**Movie EV1, related to Figure 3. Syn-TeLC expression desynchronizes circadian rhythms of PER2::LUC, while leaving Syn-GABASnFR oscillations intact.** Representative multiplexed time-lapse movie of an SCN slice co-expressing PER2::LUC (magenta) and Syn-GABASnFR (green) before and after Syn-TeLC transduction, showing desynchronization and reduced amplitude of PER2::LUC rhythms, and unperturbed rhythms of Syn-GABASnFR.
