# Supplementary material for: Rhythmic astrocytic GABA production synchronizes neuronal circadian timekeeping in the suprachiasmatic nucleus
Source: EMBO J. 2024 Dec 2;44(2):356–81. doi: 10.1038/s44318-024-00324-w (PMC11731042; doi:10.1038/s44318-024-00324-w)
Supplement: Supplementary file 4 — Movie EV2 [file 44318_2024_324_MOESM4_ESM.zip › MovieEV2_zip/MovieEV2_legend.docx]

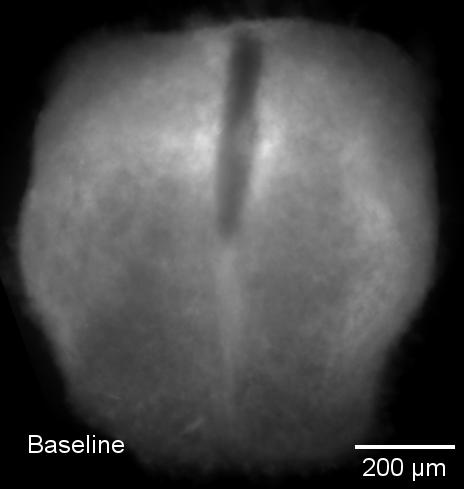


**Movie EV2, related to Figure 5. MAO-B inhibition abolishes circadian rhythms of extracellular GABA.** Representative time-lapse movie of an SCN slice expressing Syn-GABASnFR before and after treatment with Selegiline, showing abolition of GABA rhythms.
