# Supplementary material for: Rhythmic astrocytic GABA production synchronizes neuronal circadian timekeeping in the suprachiasmatic nucleus
Source: EMBO J. 2024 Dec 2;44(2):356–81. doi: 10.1038/s44318-024-00324-w (PMC11731042; doi:10.1038/s44318-024-00324-w)
Supplement: Supplementary file 5 — Movie EV3 [file 44318_2024_324_MOESM5_ESM.zip › MovieEV3_zip/MovieEV3_legend.docx]

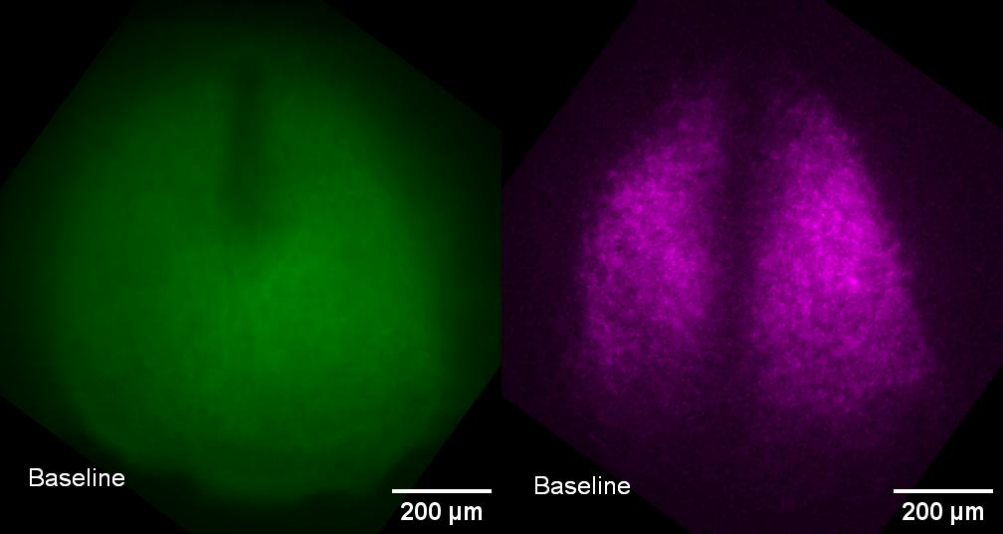


**Movie EV3, related to Figure 6 and 7. ALDH1A1 inhibition impairs circadian rhythms of extracellular GABA and weakens PER2::LUC oscillations.** Representative multiplexed time-lapse movie of an SCN slice co-expressing Syn-GABASnFR (green) and PER2::LUC (magenta), before treatment (baseline), with A37, and after drug washout, showing strong, temporary impairment of GABA rhythms, accompanied by progressive weakening of PER2::LUC, reversed upon washout.
