# Supplementary material for: Rhythmic astrocytic GABA production synchronizes neuronal circadian timekeeping in the suprachiasmatic nucleus
Source: EMBO J. 2024 Dec 2;44(2):356–81. doi: 10.1038/s44318-024-00324-w (PMC11731042; doi:10.1038/s44318-024-00324-w)
Supplement: Supplementary file 7 — Expanded View Figures [file 44318_2024_324_MOESM7_ESM.pdf]

Expanded View Figures

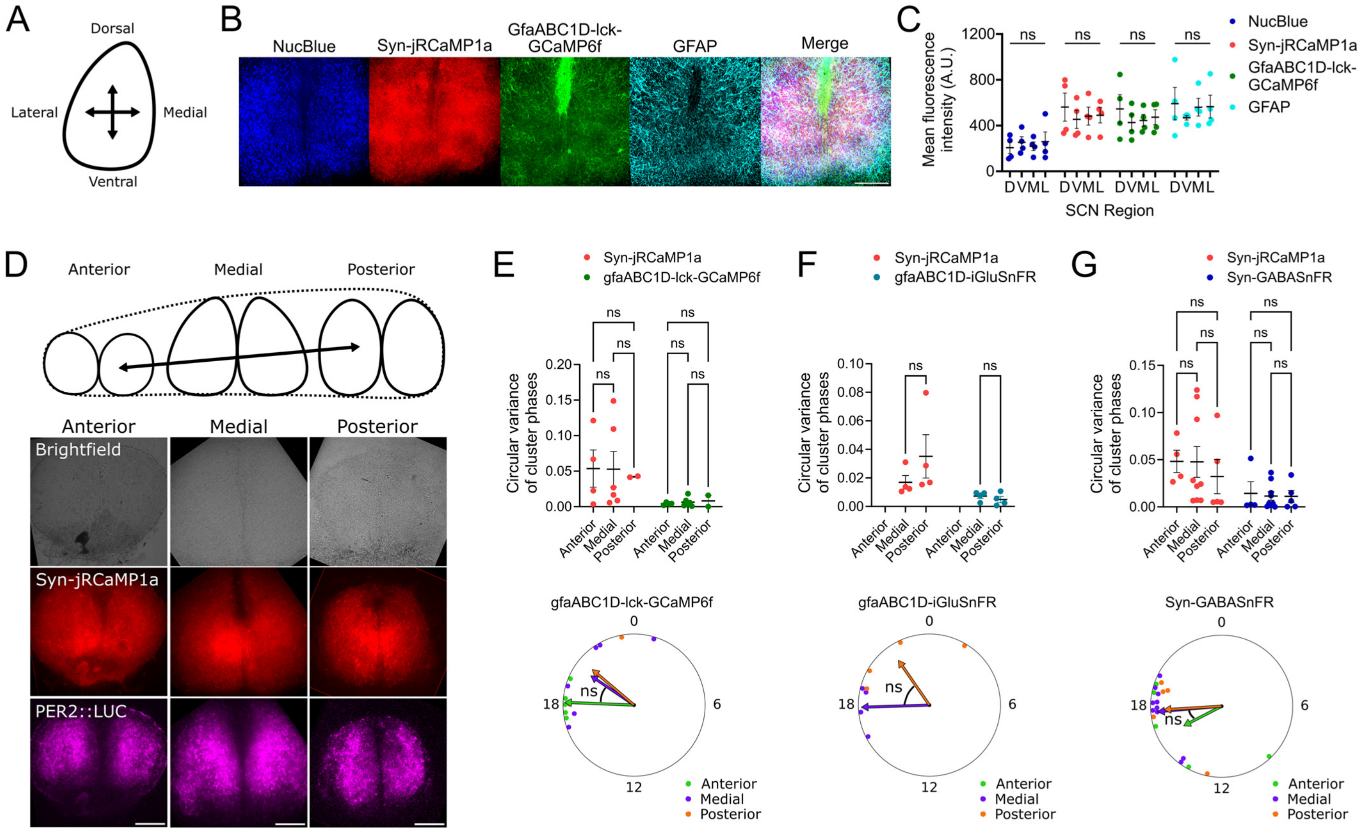

Figure EV1. Astrocytic calcium reporter is evenly expressed across the SCN, and spatiotemporal activity of astrocyte reporters is homogenous along the SCN anterior-posterior axis.

(**A**) Schematic showing spatial regions within a coronal SCN slice with dorsal, medial, lateral and ventral edges. (**B**) Representative confocal image of an SCN slice expressing neuronal (Syn-jRCaMP1a) and astrocytic (GfaABC1D-lck-GCaMP6f) calcium reporters, counterstained with NucBlue and GFAP antibody. Scale bar = 200 µm. (**C**) Quantification of mean fluorescence intensity of each reporter in the dorsal (D), ventral (V), medial (M) and lateral (L) SCN regions (see Methods), showing no detectable spatial differences in the expression of the neuronal or astrocytic calcium indicators, or GFAP staining intensity within the different SCN regions. N = 4 SCN slices, two-way ANOVA with matching and post hoc Šídák's test. (**D**) Schematic showing the shape of SCN nuclei along the anterior-posterior axis, with images of one representative SCN expressing Syn-jRCaMP1a and PER2::LUC for each region. Scale bar = 200 µm. (**E**) Top panel shows circular variance of phases across clusters in SCN slices expressing Syn-jRCaMP1a and GfaABC1D-lck-GCaMP6f divided by region across A-P axis as shown in (**D**). *N* = 2–6 SCN slices per region. Bottom panel shows Rayleigh plot of circadian phases of GfaABC1D-lck-GCaMP6f relative to co-detected Syn-jRCaMP1a (peaking at CT6) within each region across the A-P axis. Each dot represents one SCN slice, vector direction indicates mean phase, and vector length inversely indicates circular dispersion. (**F**) Top panel shows circular variance of cluster phases of Syn-jRCaMP1a and GfaABC1D-iGluSnFR by region. *N* = 4 SCN slices per region. Bottom panel shows Rayleigh plot of circadian phases of GfaABC1D-iGluSnFR relative to co-detected Syn-jRCaMP1a by region. (**G**) Top panel shows circular variance of cluster phases of Syn-jRCaMP1a and Syn-GABASnFR by region. *N* = 4–10 SCN slices per region. Bottom panel shows Rayleigh plot of circadian phases of Syn-GABASnFR relative to co-detected Syn-jRCaMP1a by region. All linear graphs show mean ± SEM, graphs in top panel of (**E–G**) show two-way mixed-effects analysis with matching and post hoc Šídák's test. Circular Rayleigh plots in bottom panel of (**E–G**) show Watson-Williams test of homogeneity of means. ns = non-significant. A detailed statistical report for this figure is provided in Appendix Table S1. Source data are available online for this figure.

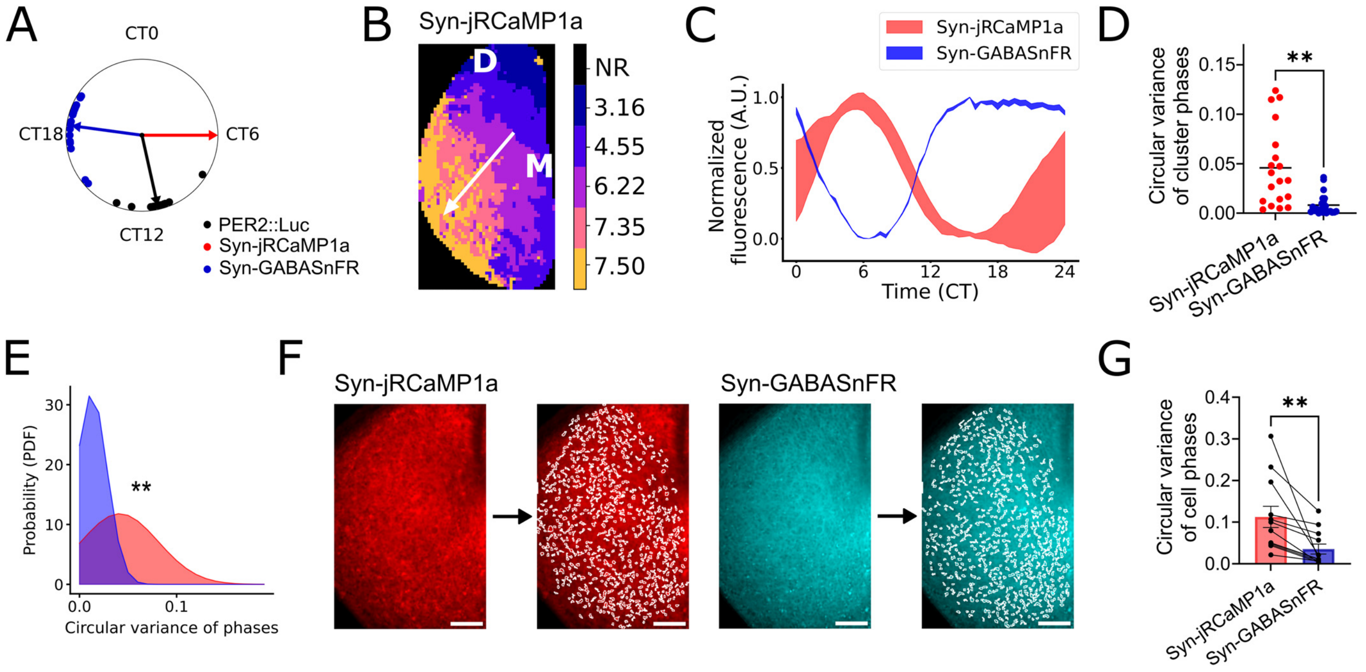

Figure EV2. Characterization of phase relationship between Syn-jRCaMP1a and Syn-GABASnFR and their synchronization across the SCN network and single cells.

(**A**) Rayleigh plot showing circadian phase of Syn-GABASnFR (blue) and PER2::LUC (black) relative to co-detected Syn-jRCaMP1a. (**B**) Representative circadian phase cluster map of Syn-jRCaMP1a, showing same SCN as co-detected PER2::LUC and Syn-GABASnFR phase map in Fig. 2C. One SCN nucleus is shown (dorsal (D) and medial (M) area indicated). Color bars indicate cluster phases, NR = non-rhythmic. White arrow indicates the direction of the phase progression. (**C**) Representative standard deviation of cluster time series of co-detected Syn-jRCaMP1a and Syn-GABASnFR. (**D**) Inter-cluster phase dispersal (measured by circular variance) of co-detected Syn-jRCaMP1a and Syn-GABASnFR (N = 19 SCN slices). Paired two-tailed *t* test, $P = 0.0013$. (**E**) PDF of cluster phase variance for each co-detected reporter, with Kolmogorov–Smirnov test, $P = 0.00397$. (**F**) Representative images of averaged Syn-jRCaMP1a (left) and Syn-GABASnFR (right) signal in an SCN slice with detected single cells indicated in white to the right. Scale bar = 100 µm. (**G**) Circular variance of circadian phases of Syn-jRCaMP1a or Syn-GABASnFR across individual cells across the SCN, each data point represents 1 SCN slice, with 200–300 cells measured per slice. Two-tailed paired *t* test, $P = 0.0076$. All graphs are mean ± SEM, $^{**}P < 0.01$. A detailed statistical report for this figure is provided in Appendix Table S1. Source data are available online for this figure.

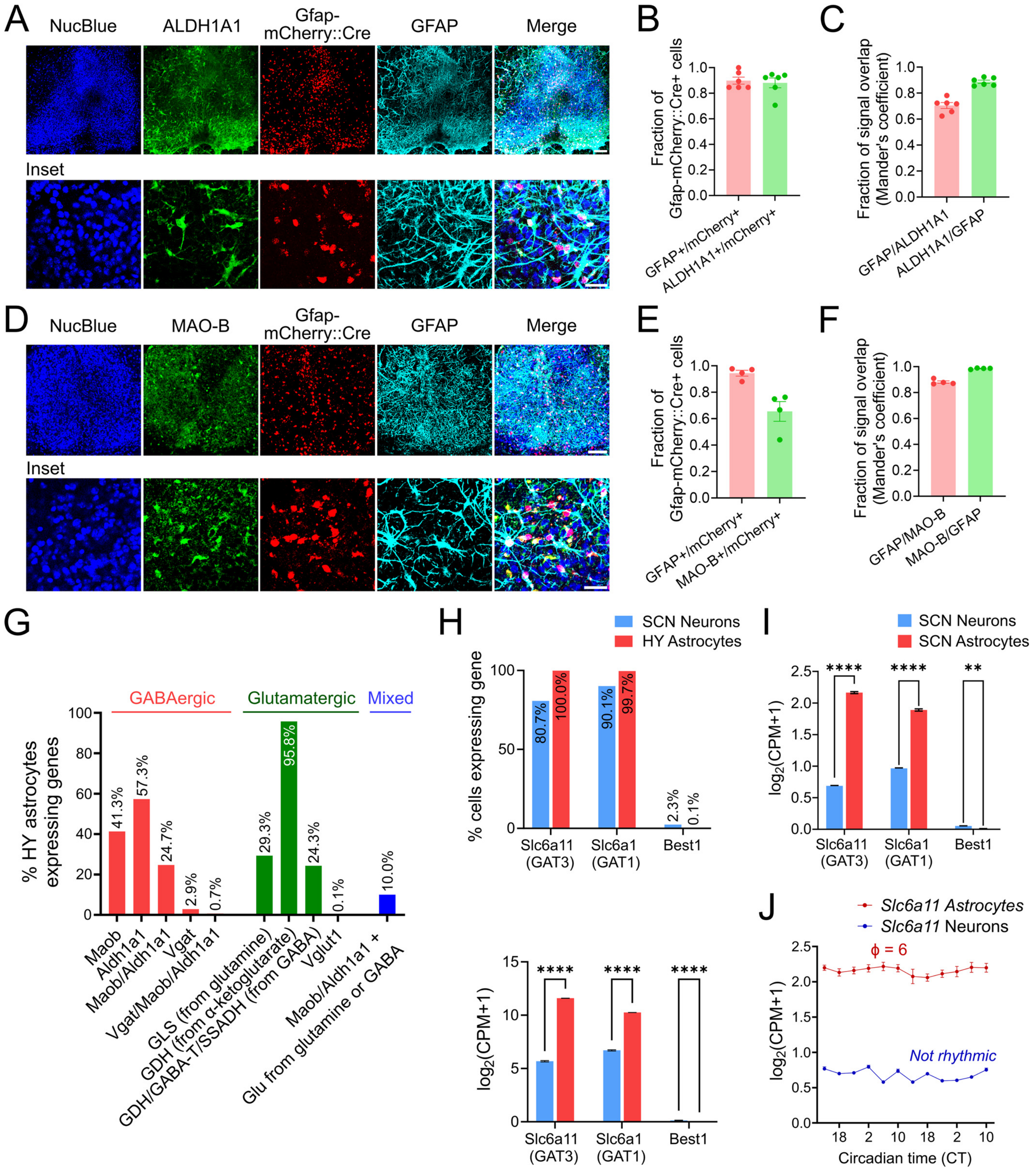
A
NucBlue
ALDH1A1
Gfap-mCherry::Cre
GFAP
Merge
Inset
B
Fraction of Gfap-mCherry::Cre+ cells
GFAP+/mCherry+
ALDH1A1+/mCherry+
C
Fraction of signal overlap (Mander's coefficient)
GFAP/ALDH1A1
ALDH1A1/GFAP
D
NucBlue
MAO-B
Gfap-mCherry::Cre
GFAP
Merge
Inset
E
Fraction of Gfap-mCherry::Cre+ cells
GFAP+/mCherry+
MAO-B+/mCherry+
F
Fraction of signal overlap (Mander's coefficient)
GFAP/MAO-B
MAO-B/GFAP
G
GABAergic
Glutamatergic
Mixed
% HY astrocytes expressing genes
41.3%
57.3%
24.7%
2.9%
0.7%
29.3%
95.8%
24.3%
0.1%
10.0%
Maob
Aldh1a1
Maob/Aldh1a1
Vgat
Vgat/Maob/Aldh1a1
GLS (from glutamine)
GDH (from α-ketoglutarate)
GDH/GABA-T/SSADH (from GABA)
Vglut1
Maob/Aldh1a1 +
Glu from glutamine or GABA
H
SCN Neurons
HY Astrocytes
% cells expressing gene
80.7%
100.0%
90.1%
99.7%
2.3%
0.1%
Slc6a11 (GAT3)
Slc6a1 (GAT1)
Best1
log2(CPM+1)
****
****
****
I
SCN Neurons
SCN Astrocytes
log2(CPM+1)
****
****
**
Slc6a11 (GAT3)
Slc6a1 (GAT1)
Best1
J
Slc6a11 Astrocytes
Slc6a11 Neurons
ϕ = 6
Not rhythmic
log2(CPM+1)
Circadian time (CT)

© The Author(s)

Figure EV3. MAO-B and ALDH1A1 protein expression in SCN astrocytes and characterization of GABAergic astrocytes.

(**A**) Representative confocal image of an SCN slice expressing Gfap-mCherry::Cre counterstained with NucBlue, ALDH1A1 and GFAP antibodies. Inset with higher magnification shown below. Scale bar = 100 µm (top row), 30 µm (bottom row). (**B**) Fraction of Gfap-mCherry::Cre^{+} astrocytes co-expressing GFAP or ALDH1A1. $N = 6$ SCN slices. (**C**) Fraction of relative signal overlap, as determined by Mander's coefficient of GFAP and ALDH1A1. N = 6 SCN slices. (**D**) Representative confocal image of an SCN slice expressing Gfap-mCherry::Cre counterstained with NucBlue, MAO-B and GFAP antibodies. Inset with higher magnification shown below. Scale bar = 100 µm (top row), 30 µm (bottom row). (**E**) Fraction of Gfap-mCherry::Cre^{+} astrocytes co-expressing GFAP or MAO-B. $N = 4$ SCN slices. (**F**) Fraction of relative signal overlap, as determined by Mander's coefficient of GFAP and MAO-B. $N = 4$ SCN slices. (**G**) Characterization of potential GABA- and/or glutamate-producing hypothalamic astrocytes based on scRNA-Seq data from the Yao et al (2023) dataset. Graph shows percentage of astrocytes expressing one or more genes involved in GABA production (GABAergic), glutamate production (Glutamatergic) or both (Mixed). N = 20,549 hypothalamic astrocytes. (**H**) Top panel shows percentage of SCN neurons and hypothalamic astrocytes expressing GABA transporters *Slc6a11, Slc6a1* or *Best1* in the Yao et al, scRNA-Seq (2023) dataset. Bottom panel shows normalized gene expression levels of each GABA transporter gene. $N = 1836$ SCN neurons and $N = 20{,}549$ hypothalamic astrocytes. (**I**) Normalized gene expression levels of GABA transporters in SCN neurons and SCN astrocytes from the Wen et al, (2020) scRNA-Seq dataset. $N = 12{,}018$ SCN neurons and $N = 8{,}429$ SCN astrocytes. ****$P < 0.0001$, **$P = 0.0038$. (**J**) Time series of normalized gene expression levels of *Slc6a11*, encoding GAT3, in SCN astrocytes and neurons. $N = 12{,}018$ SCN neurons and $N = 8{,}429$ SCN astrocytes, with 238–2410 cells/timepoint. eJTK Cycle rhythmicity test with Benjamini–Hochberg correction, $P = 0.004$ with circadian peak at CT6 in astrocytes (indicated as ϕ on the plot), not significantly rhythmic in neurons as indicated. All graphs show mean ± SEM, and panels (**H**, **I**) show two-way ANOVA with post hoc Šidák's test. A detailed statistical report for this figure is provided in Appendix Table S1. Source data are available online for this figure.

 © The Author(s)

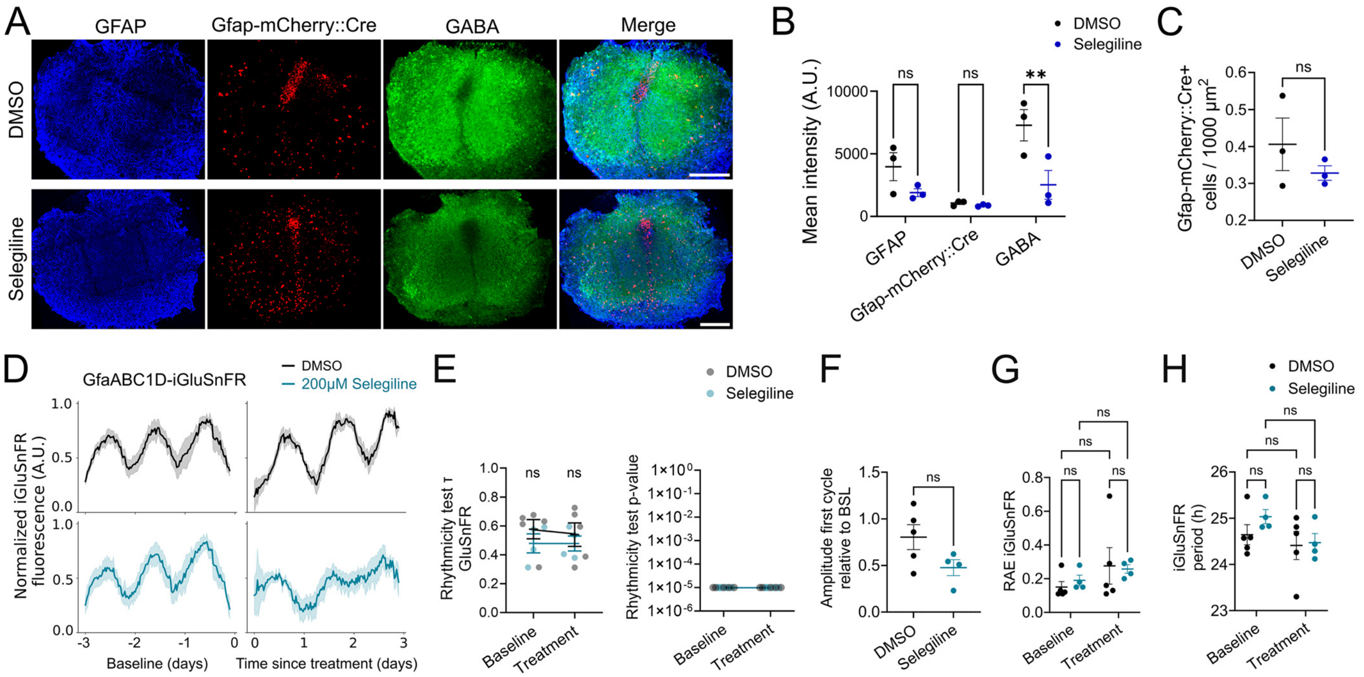

Figure EV4. Selegiline treatment decreases GABA concentration in SCN slices without significantly affecting GFAP immunoreactivity or circadian rhythms of extracellular glutamate.

(**A**) Representative confocal images of fixed SCN slices expressing Gfap-mCherry::Cre, and stained with antibodies against GFAP and GABA, 4 days after treatment with Selegiline or DMSO. Scale bar = 200 µm. (**B**) Quantification of mean fluorescence intensity of GFAP antibody, Gfap-mCherry::Cre and GABA antibody in SCN slices treated with Selegiline or DMSO. N_{DMSO} = 3, $N_{Selegiline}$ = 3 SCN slices. Two-way ANOVA with matching and post hoc Šídák's test, $P = 0.0053$ for GABA DMSO vs Selegiline. (**C**) Number of Gfap-mCherry::Cre-expressing cells per 1000 µm^2 tissue in slices treated with DMSO or Selegiline. N_{DMSO} = 3, $N_{Selegiline}$ = 3 SCN slices. Two-tailed unpaired *t* test. (**D**) Averaged, aligned time series of extracellular glutamate reporter (GfaABC1D-iGluSnFR) before and after treatment with 200 µM Selegiline (teal) or DMSO (black). N_{DMSO} = 5, $N_{Selegiline}$ = 4 SCN slices. (**E**) Left panel shows τ values obtained from eJTK Cycle rhythmicity test on time series of GfaABC1D-iGluSnFR before and within 1–3 days after treatment with Selegiline or DMSO. Right panel shows the *P* value obtained from eJTK Cycle rhythmicity test empirically calculated against random noise data. N_{DMSO} = 5, $N_{Selegiline}$ = 4 SCN slices. (**F**) GfaABC1D-iGluSnFR amplitude of first cycle of rhythms (over 30 h) after treatment with Selegiline or DMSO relative to baseline. N_{DMSO} = 5, $N_{Selegiline}$ = 4 SCN slices. Two-tailed unpaired *t* test. (**G**) RAE of GfaABC1D-iGluSnFR rhythms before and after Selegiline treatment. (**H**) Period of GfaABC1D-iGluSnFR rhythms before and after Selegiline treatment. N_{DMSO} = 5, $N_{Selegiline}$= 4 SCN slices. Graphs (**E**, **G**, **H**) show two-way mixed-effects analysis with matching, with post hoc Šidák's test. All graphs, including time series, show mean ± SEM, except right panel in (**E**) which shows median ± interquartile range due to logarithmic scale, ns = non-significant, $^{**}P < 0.01$. A detailed statistical report for this figure is provided in Appendix Table S1. Source data are available online for this figure.

© The Author(s)

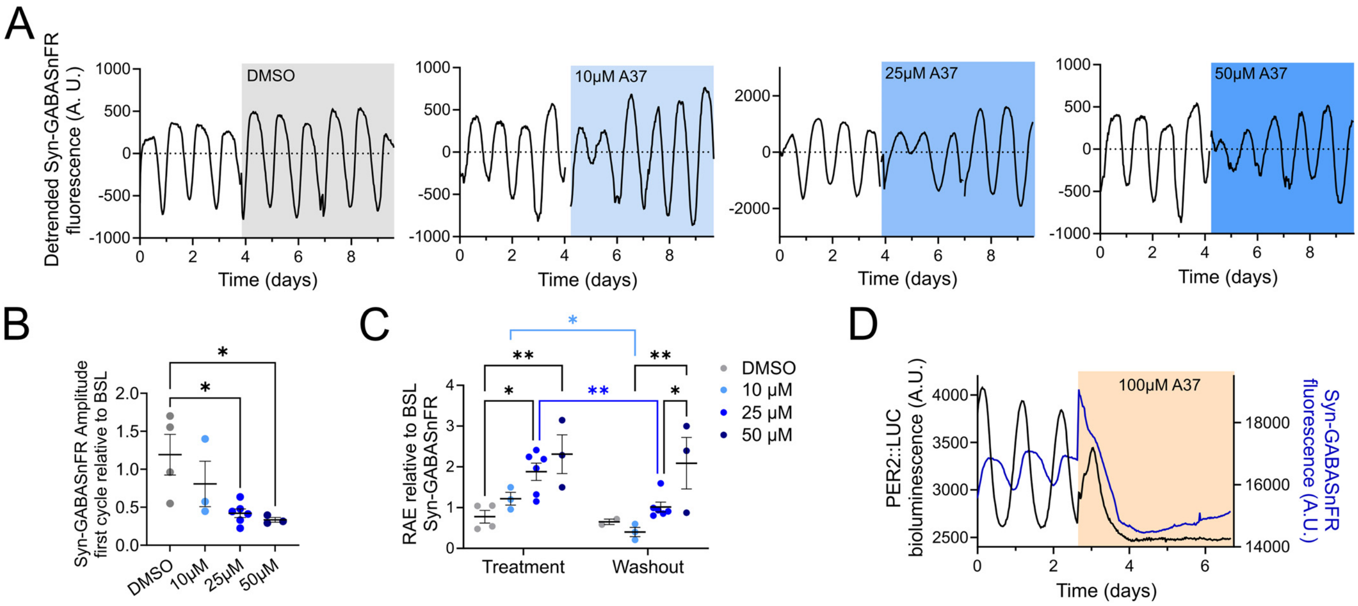

Figure EV5. A37-mediated ALDH1A1 inhibition suppresses extracellular rhythms of GABA in a dose-dependent manner.

(**A**) Representative time series of SCN slices expressing Syn-GABASnFR before and after treatment with increasing concentrations of A37 from left to right: DMSO (0 μM A37), 10 μM, 25 μM and 50 μM A37. (**B**) Amplitude of the first cycle (30 h) of Syn-GABASnFR rhythms after treatment with increasing concentrations of A37 relative to baseline. One-way ANOVA, with post hoc Tukey's *t* test shown, $P = 0.0161$ for DMSO-25μM, and $P = 0.0233$ for DMSO-50μM. $N_{DMSO} = 4$, $N_{10} = 3$, $N_{25} = 6$; $N_{50} = 3$. (**C**) RAE of Syn-GABASnFR rhythms with A37 treatment and after washout of increasing concentrations of A37 relative to baseline. Treatment: $N_{DMSO} = 4$, $N_{10} = 3$, $N_{25} = 6$; $N_{50} = 3$, washout: $N_{DMSO} = 2$, $N_{10} = 3$, $N_{25} = 6$; $N_{50} = 3$. Mixed-effects analysis with matching, timepoint effect $P < 0.01$, A37 dose effect $P < 0.01$, with post hoc Sidak's test, treatment: DMSO-25 μM, $P = 0.0206$, DMSO-50 μM, $P = 0.0052$; washout: 10 μM–50 μM, $P = 0.004$, 25 μM–50 μM, $P = 0.0477$; comparisons across timepoints are shown in the corresponding color. (**D**) Representative time series of PER2::LUC and Syn-GABASnFR before and after treatment with 100 μM A37, showing immediate tissue death. All graphs are mean ± SEM. *$P < 0.05$, **$P < 0.01$. A detailed statistical report for this figure is provided in Appendix Table S1. Source data are available online for this figure.

 © The Author(s)
